# A Myocardial Infarction Following a Mild Case of COVID-19 in a 26-Year-Old Male

**DOI:** 10.7759/cureus.27026

**Published:** 2022-07-19

**Authors:** Ryan M Wolsky, Cinthia T Bateman

**Affiliations:** 1 Internal Medicine, Advocate Christ Medical Center, Oak Lawn, USA; 2 Cardiovascular Medicine, South Denver Cardiology Associates, Littleton, USA

**Keywords:** myocarditis, pericarditis, outpatient, covid-19, covid, coronavirus, sars-cov-2, hypercoagulability

## Abstract

A well-known complication of COVID-19 is hypercoagulability in both the venous and arterial circulation. Most cases of hypercoagulability-related complications have been described in hospitalized patients with severe diseases and multiple comorbidities. However, this report outlines a case of myocardial infarction in a young patient with no prior medical history after only a mild course of COVID-19. His symptoms resolved after a mild 12-day illness course that did not require hospitalization or supplemental oxygen. Three days after the resolution of his symptoms (15 days after testing positive), the patient presented to the emergency department with crushing chest pain and was found to have complete thrombotic occlusion of his left anterior descending artery. Hypercoagulability in COVID-19 patients is suspected to be caused by vascular endothelial injury and cytokine storm. This has been demonstrated in the arterial and venous circulation, as seen in histopathology samples as well as increased incidence of acute limb ischemia in COVID-19 patients. Additionally, COVID-19 is known to have myocardial involvement, as demonstrated by elevations in cardiac enzymes and cardiac imaging findings that may persist months after initial infection. Those affected by COVID-19 may have dangerous cardiovascular complications that persist after the resolution of the acute viral illness.

## Introduction

The COVID-19 pandemic has left a lasting impact on the world. It has infected more than 359 million people worldwide, resulting in more than 5.6 million deaths [1]. In the time that has passed since the first known cases were reported in late 2019, the scientific community has learned a great amount about this new virus. However, there is undoubtedly more to discover.

There have been many documented cases of COVID-19-related hypercoagulability syndromes, leading to deep venous thrombosis, pulmonary embolism, and more [2-4]. However, this report outlines a dramatic case of myocardial infarction in a young patient, with no prior medical history other than class 1 obesity, taking no medications, after only a mild course of COVID-19. As COVID-19 continues to propagate throughout the world, it is important to be aware of possible syndromes associated with infection. This would enable healthcare workers to prepare for such adverse outcomes and better optimize patient care during both the acute infection phase and the post-infection phase.

## Case presentation

A 26-year-old male tested positive for COVID-19 after complaining of loss of smell, fever, cough, and upset stomach. The patient had a past medical history that was only significant for class 1 obesity. He had no history of diabetes, renal disease, hypertension, or heart disease. He had no family history of hyperlipidemia, myocardial infarction, or hypertension. He worked at a retail store, exercised several times per week, and did not take any daily medications prior to his infection. His symptoms resolved after a mild 12-day illness course. He did not require hospitalization, supplemental oxygen, or medical therapy for the management of his acute COVID-19 disease.

Three days after the resolution of his symptoms (15 days after testing positive), the patient presented to the emergency department with waxing and waning crushing chest pain. He did not report to the emergency room until seven to 10 hours after symptom onset. The patient was diaphoretic, restless, and pale. His temperature was 97.9°F, pulse was 110 beats per minute, respirations were 24 per minute, and blood pressure was 171/98 mmHg.

**Figure 1:**
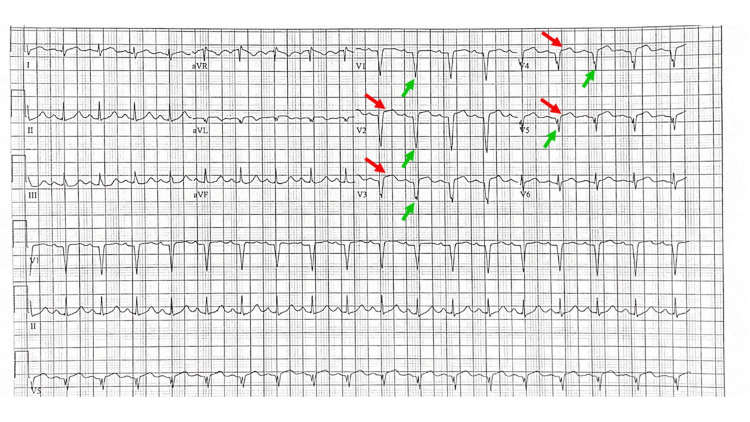
The patient’s electrocardiogram shows sinus tachycardia (130 beats/min), ST-segment elevation in leads V2-V5 (red arrows), and pathological Q waves in leads V1-V5 and aVL (green arrows).

A 12-lead electrocardiogram was performed, which revealed anterior wall ST-segment elevation myocardial infarction with reciprocal changes in the inferior leads (Figure 1). Lower extremity Doppler ultrasound was negative for deep venous thrombosis. The patient was taken to the cardiac catheterization lab where he was found to have complete thrombotic occlusion of his left anterior descending artery. Catheterization also revealed a left ventricular apical thrombus. A drug-eluting stent was placed, then the patient was placed on dual antiplatelet therapy with ticagrelor and aspirin. The patient was also placed on apixaban for the resolution of the apical thrombus.

An echocardiogram on hospitalization day two revealed an ejection fraction of 27% with severely reduced left ventricular systolic function and global hypokinesis. Labs drawn on the same day revealed elevated triglycerides and low-density lipoprotein (230 mg/dl and 113 mg/dl, respectively). Total cholesterol and high-density lipoprotein were within normal limits. A repeat echocardiogram on Day 92 revealed mild improvement in ejection fraction (30% to 35%). The apical thrombus had resolved, however, there was still mid-distal septal and apical akinesis.

## Discussion

Hypercoagulability is a well-known complication of COVID-19 disease that leads to both primary thrombosis in the pulmonary circulation as well as emboli originating from non-pulmonic sources [2-5]. In a study of 3334 patients hospitalized for COVID-19 by Bilaloglu et al., 533 patients had thromboembolism (16%), and 298 patients (8.9%) suffered from myocardial infarction [4]. Although this phenomenon has been well-documented in hospitalized patients, it is less common in patients without risk factors following a course of only mild COVID-19.

Vascular endothelial injury and cytokine storm are key contributors to thrombogenesis in COVID-19 patients. The virus infects host endothelial cells by binding to the angiotensin-converting enzyme 2 (ACE2) protein [5]. Histological analysis of pulmonary tissue in COVID-19 patients revealed disruption of intracellular junctions, cellular swelling, and loss of basal membrane integrity of cells that stained positive for the SARS-CoV-2 virus [5]. Separately, cytokine storm is also a major contributor. Damage to microvasculature as described above increases tissue factor expression on endothelial cells and macrophages causing the subsequent release of tumor necrosis factor alpha, interleukin-1, and interleukin-6. These pro-inflammatory cytokines have all been found at significantly higher levels in the serum of COVID-19 patients, where they activate the coagulation cascade and lead to microvascular thrombosis [6].

Coronavirus disease may cause hypercoagulability manifestations in both the venous and arterial circulation [3,4,7]. In the single-center retrospective cohort study by Bellosta et al., a large increase in the incidence of acute limb ischemia (ALI) from the months of January to March of 2020 was attributed to COVID-19 infection [7]. Bellosta et al. observed a gross morphological difference between COVID-19-induced arterial thrombi when compared to typical non-COVID-related specimens. The group also observed the demographics of patients diagnosed with ALI was different than typical non-COVID-related ALI. Typical ALI affects young female patients; however, the group found patients in this study were both young and old, with a male predominance [7]. These findings suggest that COVID-19 disease may cause both arterial and venous thrombosis, which increases the likelihood of COVID-19-induced myocardial infarction.

It has been well-established that the SARS-CoV-2 virus has cardiovascular involvement, as suggested by the less-favorable outcomes for patients with pre-existing cardiac conditions, as well as elevated troponin, electrocardiogram (EKG) changes, and echocardiogram abnormalities in the setting of COVID-19 disease [3,8,9]. Important to note is that patients may have cardiac involvement with or without the presence of respiratory symptoms [3,8]. A proposed mechanism for possible perimyocardial involvement involves SARS-CoV-2 entering cardiac myocytes and pericytes, which causes subsequent diffuse myocardial edema, ventricular dysfunction, as well as possible pericarditis, pericardial effusion, and tamponade [3].

Although this patient did not have any known past medical history or take any medications prior to his infection, he did have class 1 obesity (body mass index (BMI) 30-34.9 kg/m^2^). Association between obesity and death in patients with COVID-19 was analyzed in two separate studies by Tartof et al. and Anderson et al. Both studies noted a statistically significant difference between obese (BMI >30 kg/m^2^) and non-obese (BMI 18.5-24.9 kg/m^2^) patients when analyzing death rates (relative risk >3 and hazard ratio 1.6 in each respective study). However, this conclusion was not supported when patients were further stratified to classes of obesity. There was no significant difference in death rates between patients with class 1 obesity and normal BMI in either study: risk ratio 1.26, confidence interval (CI) 0.82-1.95), and hazard ratio 1.0 (0.8 to 1.2), respectively. This was illustrated by the patient in this report, as he did not require supplemental oxygen, hospitalization, or medical therapy for his COVID-19 disease [10,11].

It has also been documented that cardiac inflammation may last well-beyond the acute COVID-19 disease process. In a study completed by Puntmann et al., labs were collected and cardiac magnetic resonance imaging (CMRI) was completed on 100 post-COVID patients (mean follow-up time 71 days, 33 patients requiring hospitalization) [9]. Of this study population, 78% of patients had some sort of cardiac abnormality, consisting of decreased left ventricular/right ventricular ejection fraction, higher left ventricle volume, or raised native T1 and T2 measures [9]. The most common finding was pericardial inflammation (60%), defined by abnormal native T1 or T2 measurements. These findings not only support that cardiac inflammation lasts long after the resolution of COVID-19 symptoms, but it also further supports that COVID-19 disease often has perimyocardial involvement [9].

Additionally, a 2018 study by Kwong et al. discovered a statistically-significant six-fold increase in the rate of myocardial infarction during the immediate resolution phase of influenza virus infection [12]. These findings support the argument that infection may precipitate acute coronary syndromes through the increase in endothelial dysfunction, increase in metabolic demand, hypoxemia, and hypotension. As COVID-19 and influenza virus both commonly cause pneumonia-like disease manifestations, these findings may also support the argument that COVID-19 increases the risk of myocardial infarction [12].

## Conclusions

As demonstrated in this case, COVID-19 increases the risk of hypercoagulability and related thrombosis and may cause myocardial infarction, regardless of disease severity. The effects of COVID-19 last long beyond the acute stage of disease, and may cause lasting cardiac dysfunction. In addition to the well-known pulmonic manifestations, patients with mild-to-moderate disease courses may still have dangerous cardiac complications as well.
